# High resolution physical map of porcine chromosome 7 QTL region and comparative mapping of this region among vertebrate genomes

**DOI:** 10.1186/1471-2164-7-13

**Published:** 2006-01-24

**Authors:** Julie Demars, Juliette Riquet, Katia Feve, Mathieu Gautier, Mireille Morisson, Olivier Demeure, Christine Renard, Patrick Chardon, Denis Milan

**Affiliations:** 1 Laboratoire de Génétique Cellulaire, INRA, BP52627, 31326 Castanet-Tolosan, France; 2 Laboratoire de Génétique Biochimique et de Cytogénétique, INRA, 78352 Jouy en Josas, France; 3 Laboratoire de Génétique animale, INRA, 35042 Rennes, France; 4 Laboratoire de Radiobiologie et d'Etude du Génome, INRA-CEA, 78352 Jouy en Josas, France

## Abstract

**Background:**

On porcine chromosome 7, the region surrounding the Major Histocompatibility Complex (MHC) contains several Quantitative Trait Loci (QTL) influencing many traits including growth, back fat thickness and carcass composition. Previous studies highlighted that a fragment of ~3.7 Mb is located within the Swine Leucocyte Antigen (SLA) complex. Internal rearrangements of this fragment were suggested, and partial contigs had been built, but further characterization of this region and identification of all human chromosomal fragments orthologous to this porcine fragment had to be carried out.

**Results:**

A whole physical map of the region was constructed by integrating Radiation Hybrid (RH) mapping, BAC fingerprinting data of the INRA BAC library and anchoring BAC end sequences on the human genome. 17 genes and 2 reference microsatellites were ordered on the high resolution IMNpRH2_12000rad _Radiation Hybrid panel. A 1000:1 framework map covering 550 cR_12000 _was established and a complete contig of the region was developed. New micro rearrangements were highlighted between the porcine and human genomes. A bovine RH map was also developed in this region by mapping 16 genes. Comparison of the organization of this region in pig, cattle, human, mouse, dog and chicken genomes revealed that 1) the translocation of the fragment described previously is observed only on the bovine and porcine genomes and 2) the new internal micro rearrangements are specific of the porcine genome.

**Conclusion:**

We estimate that the region contains several rearrangements and covers 5.2 Mb of the porcine genome. The study of this complete BAC contig showed that human chromosomal fragments homologs of this heavily rearranged QTL region are all located in the region of HSA6 that surrounds the centromere. This work allows us to define a list of all candidate genes that could explain these QTL effects.

## Background

Chromosome 7 contains a region of the porcine genome in which the highest number of quantitative trait loci (QTL) have been mapped, affecting most notably growth, fat deposition and carcass composition [1-5]. Among all QTL detected on this chromosome, many have been mapped in an interval spanning the SLA complex in position 7p12-q12. Development of a high resolution porcine gene map and comparison with the human map is a major step towards the identification of the genes responsible for QTL effects. The development of two radiation hybrid panels IMpRH and IMNpRH2 constructed after irradiation of porcine cells at 7.000 and 12.000 rads [6,7] provides tools of choice for such comparative mapping studies at 2 complementary resolutions : 1) one breakage could be observed on at least one hybrid of the panel for markers at an average distance of >50 and 20 Kb, 2) the order of a set of markers can be unambiguously determined, provided that the distance between adjacent markers range from 0.25 to 1.5 Mb on IMpRH panel and from 100 to 600 Kb on IMNpRH2 panel [8].

At first glance, the chromosomal region in which QTL have been mapped on SSC7 seemed identical to the p arm of HSA6 [9,10]. A more detailed study showed that a fragment of ~3.7 Mb found at a pericentromeric location on HSA6 is situated at 23 Mb from the expected location on the porcine genome, precisely in the region of interest [11,12]. This fragment was defined by three genes (*FLJ10775*, *BMP5 *and *BAG2*) located close to the centromere at 56 Mb on HSA6p12.1-6p11.2 and mapped on SSC7q11 close to *RAB2L *and *DAXX *genes localized at 33 Mb on HSA6p21.32. A preliminary physical map of this region was proposed by Barbosa [12], but 4 limited BAC contigs were built in this rearranged region. However, it failed to establish a complete comparative map of this region and to ensure that no additional fragments found elsewhere on the human genome are located in the porcine QTL region.

We now report the building of a complete BAC map covering the whole region. This was achieved by taking advantage of the fingerprinting of a 5.5× BAC library (Chardon et al, in preparation), undertaken to establish primary BAC contigs. We monitored the assembly of the complete contig by mapping genes and markers on the high resolution IMNpRH2_12000 _panel, as previously done in the Halothane region [8]. Sequence comparison of BAC ends from this contig with the human sequence allowed us to identify 4 BAC containing the four synteny breaking points detected in this region. A local bovine RH map was also established in this region to determine if the rearrangements observed on the pig genome are specific of suines or if they occurred in an ancestral species. A multispecies comparison of this region was finally performed.

## Results

### Development of a preliminary porcine gene map

To study the complete rearrangements that occurred in this region of an estimated 3.7 Mb and containing the 3 genes *LANO *(previously called *FLJ10775*), *BMP5 *and *BAG2 *[11,12], it was necessary to: 1) define the chromosomal blocks arranged differently between HSA6 pericentromeric region and SSC7; 2) determine if additional human fragments moved in this porcine region. In the first approach, we increased the density of genes mapped in this region of the IMNpRH2_12000 _panel [7]. Using the ICCARE browser [13], we identified genes for which porcine EST were available. We chose 5 genes located on HSA6p21.32 and 5 genes located on HSA6p12.1-6p11.2: *COL11A2 *(33.24 Mb), *HSD17B8 *(33.28 Mb), *RING1 *(33.29 Mb), *VPS52 *(33.33 Mb), *RPS18 *(33.35 Mb), *GCLC *(53.47 Mb), *TINAG *(54.28 Mb), *HCRTR2 *(55.15 Mb), *COL21A1 *(56.03 Mb) and *DST *(56.43 Mb). No gene was selected below *PRIM2A *since no porcine sequence was available for the few genes located in this region very close to the centromere of HSA6. When possible, primers were developed in the 3' UTR sequence of selected genes (Table 1). For *GCLC *only, we had to choose primers in two consecutive exons framing an intron. Markers developed for these 10 genes, as well as markers previously developed [11,12] for *RXRb *(33.27 Mb), *ELOVL5 *(53.28 Mb) and *PRIM2A *(57.29 Mb), were analyzed on the 90 clones of IMNpRH2_12000 _panel. In addition to markers produced in the frame of this project, we included in the analysis markers already mapped in this region on IMpRH and IMNpRH2 panels, in particular *HKE4 *(33.28 Mb), *RAB2L *(33.37 Mb), *LANO *(53.77 Mb) (called previously *FLJ10775*),*BMP5 *(55.73 Mb) and *BAG2 *(57.15 Mb) [11,12]. An average retention frequency of 40% was observed, ranging from 27% for *PRIM2A *to 55% for *RAB2L *and *HKE4*. A draft 1000:1 framework map was then built with Carthagene software [14], using a stepwise locus adding strategy (data not shown). The robustness of the map was tested by comparison of the likelihood of the reference map to the likelihood of maps derived from the reference map by all possible permutations in a window of 6 markers and by inversion of parts of the map using the simulated annealing algorithm. The 6 additional genes (*COL11A2, RXRb, RING1, PRIM2A, COL21A1 *and *RPS18*) were mapped at their most likely location relatively to markers of this preliminary framework map, considering the distance between framework markers as fixed.

**Figure 1:**
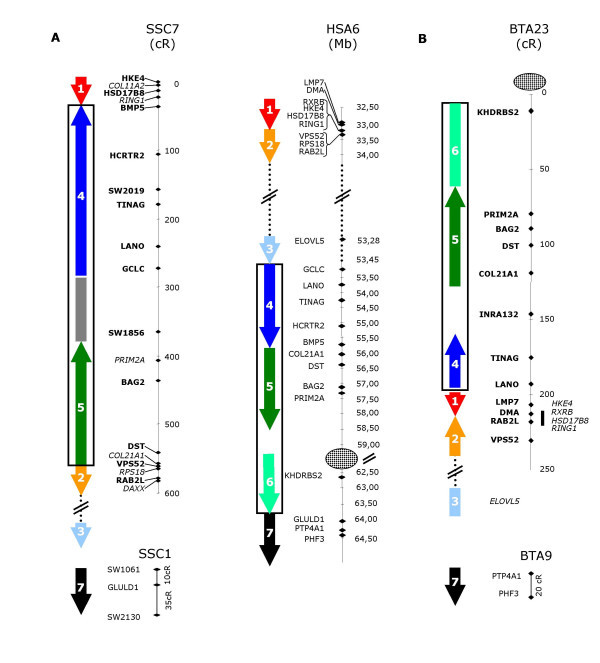
**Gene based comparative map of SSC 7q1.2 and BTA 23q11 radiation hybrid maps and HSA 6p21.32/HSA 6p12.1-q112 physical map**. A- SSC 7q1.2 radiation hybrid map was build using IMNpRH2_12000 _developed by Yerle et al [7]. B- BTA 23q11 radiation hybrid map was build using RH5000 panels developed by Williams et al [22]. The human physical map corresponds to NCBI build 35 produced by the International Human Genome Sequencing Consortium. Markers in bold on the RH map belong to the framework map, whereas markers in italic are mapped at their most likely location considering as fixed the distance between framework markers. The centromere is represented by a circle. Arrows indicate blocks of gene whose order is conserved among the three species. Hatched box on the SSC7 RH map represents a fragment where no gene mapping information is available.

To compare the order of genes between SSC7 and HSA6, we determined the location of these genes on the NCBI Build 35 assembly of the human genome available at UCSC [15]. We confirmed the insertion in pig of a large fragment between *RING1 *and *VPS52 *genes (fragments 1 and 2), which are only 40 Kb apart on the human genome (Figure 1A). The fragment found in pig between these two genes is located on human genome close to the centromere on the HSA6p arm. The mapping of these genes on the porcine RH map allowed us to distinguish two main conserved fragments: one fragment containing *BMP5 *– *HCTR2 *– Sw2019 – *TINAG *– *LANO *– *GCLC *(fragment 4), and a second fragment containing *DST*, *BAG2 *and *PRIM2A *(fragment 5). The order of genes is conserved inside each fragment; however, the 2 blocks are oppositely oriented in the porcine and human genomes. A large distance is observed between *GCLC *and *BAG2*, suggesting that another fragment is inserted between them, possibly a fragment called "6" in the human genome. This additional porcine fragment is particularly interesting because it contains Sw1856, a marker mapped at the most likely location of the QTL of interest. At that step, it was not possible to determine whether the rearranged fragment includes a short fragment located on the q arm of HSA6. The first gene found on HSA6q arm also mapped on the porcine genome, being *FLJ13159 *located on SSC1 [11].

**Table 1:** Porcine markers developed for RH mapping and BAC contig construction

**STS-EST**	**Sequence up**	**Sequence on**	**Size of PCR fragment**	**% Retention on IMNpRH2**	**Position (cR)**	**N° INRA Contig**	**Human Location (Mb)**
***Arrow number 1 on figure 2***							
bI0149A04F	CTCTCCTTCAAAGGCCTCCT	ATGCCTCAGGCGATCTAATG	233				33.03
*COL11A2*	CTCCCACCTGGAGCCTGT	CAGTGTCCTTTCTCTTCTCTTCC	154	48%			33.24
*RXRB*	TCCAGAGTCCCCTCTCACAC	CCAAATACCACCTCCACCAC	247	41%			33.27
**HKE4**	CCGCTCTCTGCTCCAGAT	AAGAAAGGCGACAATTCCAC	487	42%	0		33.28
**HSD17B8**	CTCAAGGACCCTGGACTCTG	GGAATTTATTAGGGTCACAAGCA	186	45%	15		33.28
bI0392F02R	AAGATGCCCTTGAGCTGCTA	GCAGCAGCACATTGAGAGAA	136				
*RING1*	GCCACTTGTTCTCATTTGTGTC	ATCCAAAGTGACCCCACAAG	150	48%			33.28
***Arrow number 4 on figure 2***							
bI0149A04R	CTTGGATCAAACCCACATCC	CGGCGTGTAGTGATTGCTTA	282				55.93
bI0248H04R	AGGGAATGACAACAGCAACC	TGGAGCAGATTTAGGATTTGG	163			5174	55.80
bI0123E02R	CCGAGTGGAATCATATGCTCTT	TCCTCGACGAAGTTCTCCAT	153			5174	55.78
bE276E04SP6	GTAGGGTTAGCCTGCTGTGG	TGTCGAAACTTGGCCTCTCT	127				55.77
**BMP5**	ACAGCAAAAAGCAAAGACCA	GTAAGTAGGTTGTCAGGCTTGC	230	41%	32		55.73
**bI0895F12R**	GGCAAAGCAATTCCACAGAT	CCGAAACCTCATGGTTCCTA	198	33%	59	5174	55.71
bI0123E02F	GAGACCCAGAAACCAACCAA	GCAGGCTTGTCTCCAATGAC	249			5174	55.67
bI0474F10F	CAAGTGAAGCAAATCCACCA	ACTCCTGCACTGGCCATATT	232				
bI0474F10R	CATGCTTGCTAACACCAGGA	AGTGGCTCTCAGCCTCAAAA	176				
**bI0187D03R**	AGAAACACCAGCCTCCTCAA	CCACAGCACAGTGCAGAAAT	256	43%	82	5488	55.51
bI0187D03F	CGACACCCACAAAGGAAAGT	GTGATGCATTTTGCCACATC	169			5488	
bI0773C07R	AGAAAGGTGGGGAGAGCATT	GCACTGGAGCACAGCAAATA	132				55.36
**bI0136E09F**	CTGCTTGTGGGGATACCATT	TCCTCACCTGATTCGCTACA	158	35%	89	5488	55.30
bI0773C07F	GGCATGTTGAAAGCCAAGAT	TTTGGCTTAAGGGAATGTCG	213				
bE0164A10SP6	GCACCTGGACTGGAGGAATA	AGGGCCTAGGTCCACTCAAT	115				55.23
**HCRTR2**	TGTGTCGGTGTCTGTGTCTG	CGATCCAGATGATGACGATG	131	44%	101		55.15
bE0164A10T7	AATCTTTGGGCAGTGAGTCG	CATGGAATCACCGAAATGTG	230				55.01
**bI0759D02R**	TTTTAACGCAATCTGCTTGG	CAGCTCCCTCATTTTGAAGC	151	45%	121	6382	54.90
bI0699B10R	TGTTGGTTTGGGTGTTGAGA	TCTTGAGGCAGTGTGCATTC	262			6382	
bE0047O02T7	CTCACCTCCATCCACAACCT	GCTGGTGCCAAGATCCTAAG	235				54.83
RPCI44_0470A18F	CCTAAGTGCCATTCTCCTCCT	AAAGCAGGCCACTCGAAGTA	241				54.82
**bI0131F01R**	CACCACACACAGCAACATCA	TCAGGAGCTCCCAGGAATAA	103	41%	138	1178	
bI0131F01F	CCTCTCTGGTCTCTGCATCC	CTCCTGTCTTCCAGCTCCTG	157			1178	54.78
**SW2019**	ATGATGCGAACCTGGAACTC	TATGTGTAACTTGGTCCCATGC		30%	153		
bI0444G09F	GGCAATCATTTCCACCTTGT	AGAACTGGAGGCAGTGGAGA	231			1178	
bI0704C03F	CGGCCCTATTTTTCACTTCA	CTCCCTGGTGCCAAATAGAA	172			1178	54.55
bI0295F03F	CCCACCCAAGGTTGACTAGA	GGAACTCCCAACGTGAACTC	129			5990	54.41
*bI0704C03R*	AACCCAGGACCCAAGGATAC	ACTCTCCTTCCACAGCCAGA	232	33%		1178	54.31
**TINAG**	ATTCCTGGGGAAAGTCATGG	AAGGCATGACCTTATGGAAAT	157	33%	175		54.28
bI0295F03R	GTGGTGCAGAGGGTTAAGGA	TAGGGCTTTGCTTGTCTCGT	269	47%		5990	
bI0716B06F	TCCATTTGTTGGGGGTTTTA	ATAAGCCTCAGCCTCCATGA	177			5990	54.19
**bI0355A07F**	GGCCAGATGGAAATCTTTGA	CCAGCGGATGTGTCCTATTT	252	29%	196	3302	54.17
bI0615F10R	TCTGTTTCCCATTTGCACCT	CCACTGCAGTCAAGGTCTCA	186			3302	0.00
*bI0872D06F*	TCATGCTGGCAAATGGTAAA	ATTTCTGGCAGAGCGGATAA	116	33%		3302	53.78
bI0438F07R	CGGGTGGAAGGAGATCACTA	TTCAAAGAAGGCATGGTTCC	244				53.77
**LANO**	GGGATGCTGAATGTGGTATTT	TTTTCTCTTTTTGTGCTTTTCTG	287	38%	235		53.77
bI1044F02R	TACATGCACAGTTGGGGAAA	ACCCCTTTGGTGGTGTTGTA	144				
bI0547G12F	ATCTCTGAGCAGGTCGCAGT	ATCTTCCCTGATCCCCACTT	188			2270	
bI0547G12R	TGTCAGGGTGTCGGTTGTAG	GTCCTAACCCATGCAGCAGT	174			2270	
bI0285E03R	AATTCCTCCGCTCACTCTGA	TCAGGAGGGGAAGCTGACTA	174			2270	
**GCLC**	ACCATCCTACCCTTTGGAGA	TTTCCCCCAGTAAAGACGTG	150	37%	268		53.47
**bI0879H07F**	CTGGACGTGGAGTCTGGTTT	TTGCCCTTGATGCTAAATCC	300	38%	324	2270	
***Arrow number 6 on figure 2***							
bE251O22T7	TGGCAGCTTGACATCAGTTC	AATGTGCCAGAGGGTTTGAG	210				62.48
bI0879H07R	GAGCCTGTTAGCGCATTTCT	ATGCCAAACCATCTGTCCTC	189			2270	62.51
bT79E18SP6	TGTGGTATGAACCCAGGACA	AAGGGGATTTTCCCCATGTA	211				62.60
bE235D14SP6	TCGGAAGATTGCCTATTTGG	TGAAGATGACTGCTGCCTTG	192				62.67
bE251O22SP6	ATCTGGCCCCTGTTCTCTTT	GAAAATTTCTCTCCCCTGTGC	232				62.73
bI0052D02F	ACAGCATTATGCCGATTTCT	CCTACAAGAACCTTACAGCATTAGA	100			137	62.78
bI0052D02R	TGGCTTTATAATTATGTGCTAATGAC	CCTACATGGGAAAAGAATTTGAA	169	27%		137	
bI0137B12F	GCATGGAGTTTGGTGTTGTG	TTAGGTGAAAGGCCTGATGG	165			137	
bI0137B12R	CCAAACCCAAACTCCCTGTA	TCAGCAACATGGATGCAACT	218			137	
*bI0666E01F*	GCATGCAAAGGCACTCAGTA	CAAGAGCAAATGCATTCCAA	171	35%		236	62.91
*bI0455F01R*	CAAGAGCAAATGCATTCCAA	GCATGCAAAGGCACTCAGTA	171	35%		137	62.91
bI0415F05R	TCTGAGCTGCATTTGTGACC	GCTGGTTCTGAATCCCCATA	249			236	62.98
bI0688H06R	GCATCTTTGCATGAGCCATA	CATTGGCTAGAGAGAAGAGAAACA	189			236	63.02
bI0666E01R	CCTCCGGACACTACTTCCAA	CAAAGACACACGCTGAGGAA	221			236	63.05
bI0374A03R	CAAATGCCAGGTGGTGAAAT	CCATTTTCTGAGAAGGGGTCT	192			236	
bI0415F05F	AGCAATGGAAAAGCAACTGG	CGGGAACTCCTCTGCTGTT	212			236	
bI0688H06F	ATGTTTGCACATTATGCCTTT	ACTGCAGGCCATTCTGACTT	234			236	63.67
**SW1856**	TTGTATGGTATCCTGTGATGCC	TCATTCCAAACACACAGAGTCC		42%	361		
bI0586C11R	GCTGAGTTGATGCTTGACCA	CCCACTTGGGCAAAGTAAAG	193			236	63.70
bI0222C06F	TCCCGACTACAGACCAAAGG	GCATTCGATAGCAATTTGGAA	199			236	63.70
bI0374A03F	TGGGTGCAAAAAGTTTCCAG	AGAGCGACCCAAGAAAGTGA	151			236	
bI0834F10R	AGGCTCCAGGCAAGAGAAG	GAGAGGAGGTTTGCCAGAGA	247			236	
bI0492A07R	GGCATTTGATGCTGACACAC	CATTCGGGGATAGCGTTTTA	153			236	63.70
bI0834F10F	AACTCTGCTTCCCAGTGCAT	TAGGCTGCAGGCCTTTTCTA	222			236	
bI0586C11F	ACCTTGGACGAAGTCCCTCT	GACATCCAGCATCAGCTCAA	207			236	
***Arrow number 5 on figure 2***							
bI0223C10R	CTTGGGCAAGGCTGATAGAC	CATTCCATAGTCGGGTGTTTTT	168			236	58.03
bI0762H12R	TGGCCCTAAAAAGACCAAAA	TGGACTGGGAAACAACTTCC	176			236	57.78
bI0492A07F	GGGCATCATCGTGTTCTTTT	GGCACAACTTTGACCTCATGT	240			236	57.69
bI0392B02R	GATGGCCTGGTGGTTAAAGA	AGTGAAAAATGCCACCAACC	180			236	57.67
bI0223C10F	TGTTCCAAAGGAAAATAGAACAAA	AAAGCAGAAGTGACCCAGCTA	227			236	57.65
bI0186F03R	GGTGCGGTCAAAAATGAAAA	TAAAAATCCAGGGCCACTCA	187			236	57.58
RPCI44_0334P20R	CCTCTGCCAGCCTCATTTAC	GCTTCAGGGAAGGGGATTAG	144				57.56
*bI0392B02F*	CTCCCTTGTGTCCACTTGCT	AGAGCACCACCCTTGAGAGA	206	32%		236	57.55
**bI0877E01R**	CTCCCCCTCCTCAGCTAAAT	TGAGGTTCACCGGATCTTTC	195	34%	412	7444	57.52
bI0877E01F	GGCCATTTGCAGTGTCTTTT	AAGCCTCAGGAACAATGCTG	164			7444	57.39
RPCI44_0334P20F	TCCCCCTTTGTTTCTTCCTT	CACGCAAATCAGAAGATGGA	195				57.38
bI0297A11R	CAGAATGCCAAGAGGGAGAG	CAGAATGCCAAGAGGGAGAG	161			7444	57.36
*PRIM2A*	CATAGCTTCGGAAAGGAAGG	CTTGGTGGATTGGTGAGGAT					57.29
**bI0297A11F**	CCACTGCCACTGACTCTTCA	TCAATGCAATCCCTGTCAAA	266		425	7444	0.00
bI0229F01F	CTGAGGGGAAGCCTAGACAG	CAGGAATACAGCCCCTACCA	227	41%		1421	57.12
**BAG2**	CGCCTGTTCTTCCGAGGT	GCATTTCTACGCCATTATTTCAAG	255	39%	432		57.15
bI0229F01R	TGCCACGGCATATATTTTCA	CTACACCAGAGCCACAGCAA	202			1421	
bI0192A07F	CCGACTAGGATCCATGAGGA	CCTTTTTAGGGCTGCACTTG	198			1421	
bI0192A07R	ACCCACTGATCGACGTTAGG	GTGGTTGGAATTTGGCACTT	209			1421	56.86
**bI0783E01R**	GAGGGCCTCTTAGATGCTGA	CACCCATAAGGCTTCCTCTG	260	38%	458	1421	
**bI0697C09R**	TTATCACTGCTGTGGCTTGG	TCCCTCCCTATTGCTTCCTT	213	35%	479		
**bI0697C09F**	ATGAGGCGAAACGAAATGAC	CAATGACTCGTGCTGGTTTG	191	25%	507	1421	56.67
bI0135F11R	CCCTCTGACAACCACCAGTT	GCAACATGGAGGGACCTAGA	163				56.68
bI0135F11F	TTGACCCTGTCGACTGCTTT	CAACACAGTGGTTGAACGTTTT	185				
bT220O06SP6	TGAGAGGCCTCGTGGATTAC	TTGGAGGTGGCTCAGAACTT	155				56.58
**DST**	TGTCAGGTTTTCTTTTGCTTGA	GGGACACGTTTTATTTCATAGCTT	106	32%	538		56.58
bI0105B03F	TCAGGGCACTTTCTGCTGTA	GCTGGGAGTTTGGGGTTAGT	183				
bI0105B03R	TCAGTTGGAGCTGAAAAACTCA	AAACCACAAAAGCCAAACCA	226				56.47
bT220O06T7	GAATGGGAGGAGGAAGTGGT	CAGCTAAAGGACGGACGAAC	228				56.44
bI0032D02F	TCTGTGTACCACCCCACTCA	CCATGGCTGGTATTTGAACC	257				56.15
bI0032D02R	AGGGGCCTGTTTACGTCTTT	CACCTTGACGTCTCATCAGC	120				
*COL21A1*	TGCATGCTTTCATTTTCCAT	TGTTCCTTAACAACGAAGCATT	204	37%			56.03
***Arrow number 2 on figure 2***							
**VPS52**	CAAGAAGCACAAGCCCAACT	GGGACCCAGCTTATCCTGA	190	39%	557		33.33
*RPS18*	CTGCAGCCATGGTAAGAGTT	GTCTTCACGACACAACACGA	350	38%			33.35
**RAB2L**	AAGTGTCATCANGTCGTGTCC	GCCCAGCGTAGCAGTAGAG	314	54%	578	33.37	

Markers in bold belong to the framework RH map, whereas markers in italic are positioned at the most likely location. Other markers have been developed in the frame of the contig building. The arrows on the side represent the fragments conserved between the pig and human genomes. Primer sequences, retention fraction, RH map location on porcine map and human location are indicated.

### Bac contig construction

**Figure 2:**
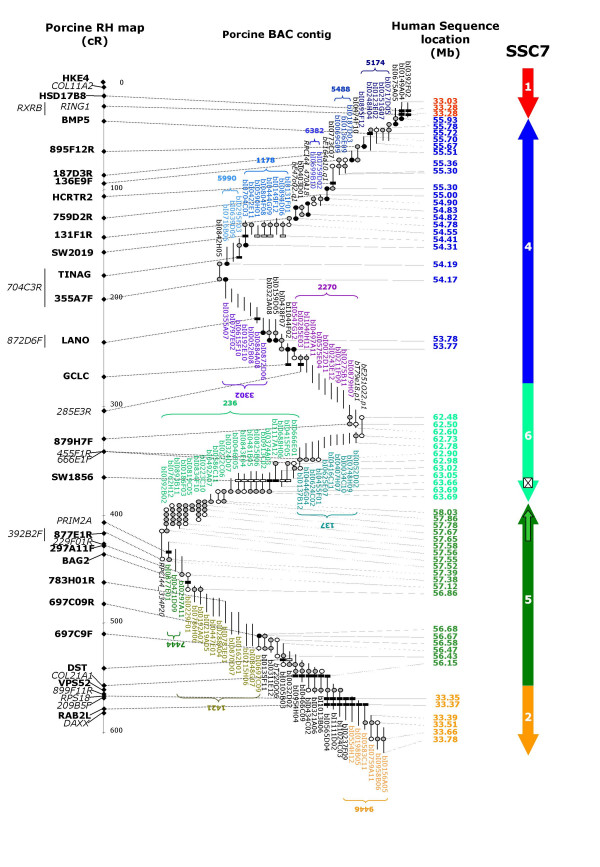
**Porcine BAC contig with anchoring on porcine RH map and on human sequence after BAC end sequence analysis**. The full contig is presented including mainly INRA BAC. Some additional BAC where recruited on the international analysis available at . The black circles represent sequenced BAC ends (BES) used for screening INRA BAC library. The white circles are BES for which a marker has been developed for a quality control of the contig by PCR on overlapping BAC (positive results are indicated with gray circles). The black boxes represent genes and microsatellites used for screening INRA BAC library, the white boxes indicate additional BAC on which a positive result has been obtained by PCR with these markers. Markers in bold on the RH map belong to the framework map, whereas markers in italic are additional markers mapped at the most likely location (when two locations are as likely, the marker is indicated on the left side of the map and its possible locations are indicated with a line). The BES for which a significant homology has been identified with sequence of HSA6 are illustrated by dotted lines. The large arrows represent the extent and the orientation of the different fragment conserved between pig and human genomes. The small arrows in fragment 5 represent the duplication identified on the human. Finally, the white box in fragment 6 is the fragment absent on the pig genome.

In order to confirm the rearrangements previously observed between HSA6 (6p21.32 and 6p12.1-11.2) and SSC7 (7q11), we built a complete BAC contig of this region. As in previous studies, an almost complete BAC contig of the SLA region has been developed [12], but a gap remained to be filled precisely in this region that was very different between the human and pig genomes. We screened by PCR the INRA BAC library [16] using primers defined for microsatellites Sw2019 and Sw1856, and for the 10 following genes *RING1*, *HSD17B8*, *BMP5, HCRTR2, TINAG, LANO, GCLC, PRIM2A, BAG2 *and *DST*. For each of these 10 genes or 2 markers (Sw2019 and Sw1856), we identified one positive BAC (Figure 2). To accelerate the establishment of a complete BAC contig, we took advantage of the availability of preliminary BAC contigs built after the fingerprinting of 72 190 BAC of the INRA library (Chardon et al, in preparation). It appeared that 8 of these 12 BAC were mapped in 8 different INRA contigs (contigs 5174, 1178, 5990, 3302, 2270, 137, 236 and 1421), containing from 3 to 21 BAC (Figure 2).

In the frame of the INRA physical mapping project, 35 000 INRA BAC regularly dispersed on the INRA contigs had been selected. The sequence of their extremities were determined at CNS (Evry, France) and compared with the human sequence (Chardon et al, in preparation). Analysis of 108 porcine BES anchored on the human region between 33 and 34 Mb, or between 53 and 64 Mb, allowed us to identify 55 BAC located on the pig genome in the chosen region. Among these BAC, 39 belonged to one of the 8 INRA contigs already identified. 8 additional BAC allowed us to identify 3 others INRA contigs (contigs 5488, 6382 and 7444) located in this same region, and the last 8 clones correspond to BAC that had not been fingerprinted.

To fill several gaps, the INRA BAC library was screened with markers derived from BES, and 12 additional INRA BAC (bI0149A04, bI0474F10, bI0403D01, bI0842H05, bI0323A08, bI0159D05, bI0438F07, bI1044F02, bI0135F11, bI0311E12, bI0105B03 and bI0032D02) were recruited. For BAC of interest whose BES had not been sequenced by the CNS, we sequenced the extremities in order to get on with the construction of the contig; these sequences were submitted to EMBL Nucleotide Sequence Database (AM156864 to AM156903). To fill the remaining gaps, we selected 7 additional BAC (bE0164A10, RPCI44_0470A18, bE0047O02, bT0079E18, bE0251O22, RPCI_440334P20 and bT220O06) on the international BAC map (Humphrey et al, in preparation, and [17] that were overlapping with BAC already selected. To check for the accuracy of the global contig, new markers were developed using BES. The overlapping of BAC derived from the different INRA contigs was tested by PCR on BAC clones (Table 1 and Figure 2).

The building of the complete BAC contig was also monitored regularly by the mapping of BAC end- markers on the IMNpRH2 RH panel, verifying that the order of the markers and the distances between them were consistent with the BAC contig results. In particular, markers found at the extremities of the 11 INRA contigs were mapped on the RH panel. For these 19 markers, an average retention frequency of 35% was observed. A 1000:1 framework map was then established, including previously mapped genes and 11 markers derived from BES. The 8 others BAC ends were mapped at their most likely location relatively to markers of the framework. This RH map covering 550 cR is in total agreement with the BAC contig covering the whole region (Figure 2).

### Analysis of BAC contig

The availability of BES from BAC located between *PRIM2A *(fragment 5) and *GCLC *(fragment 4) permitted characterization of the intermediate fragment in which no genes were mapped (Figure 2). All the significant homologies detected with these BES show that this fragment corresponds to fragment 6 located on HSA6 in the interval 57.50 – 63.5 Mb framing the centromere. Among BES anchored at this human region, bI0666E01F is highly homologous to an intron of *KHDRBS2*, demonstrating the presence of the gene in this porcine region. In addition, the comparison of porcine BES produced from BAC bI0222C06, bI0223C10 and bI0819C05 revealed that these sequences share significant homology with 2 human fragments at a distance of 600 Kb, suggesting the presence of duplication in the human genome. A dot-plot of the human region 57.73 Mb-58.9 Mb on itself was performed with Dotter, using its default settings [18]. The dot-plot showed that this region contains many large repeated sequences. A fragment of 290 Kb located at 57.74 Mb-58.03 Mb is duplicated in position 58.34 Mb-58.63 Mb, and a 50 Kb fragment from 58.07 Mb to 58.12 Mb is duplicated and turned over in position 58.73 Mb-58.77 Mb on HSA6 (results not shown). In the pig, this fragment covers ~40 cR, which corresponds to ~380 Kb suggesting that a single copy is present in the pig genome.

Finally, for BAC bI0688H06 and bI0046E05 mapped in the same region, hits obtained for the two BES of both clones were 650 Kb apart, which is not compatible with the size of a BAC. Analysis of this human region on ENSEMBL database [19] indicates that no porcine BES matches the human genome between 63.05 and 63.55 Mb, whereas BES were anchored in the surrounding region on average each 20 Kb. Altogether these results suggest that a 500 Kb region (in which no genes have already been identified on the human genome) is missing in the pig genome.

From this analysis, an estimate can be made of the size of the rearranged region on the pig genome – considering that the size of this region on the porcine genome is similar to the size of human region also found on the porcine genome. The sizes of the three rearranged fragments are: ~2.5 Mb for fragment 4, 1.8 Mb for fragment 5 and 0.9 Mb for fragment 6. The whole rearranged fragment covers thus ~5.2 Mb of the porcine genome.

**Figure 3:**
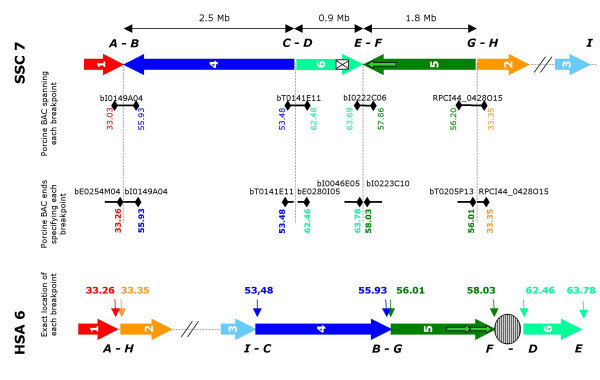
**Description of the synteny breakpoints between SSC7 and HSA6**. The arrows represent the various fragments of conserved synteny. The small green arrows symbolize a human duplication of a fragment of 290 Kb. The white box represents a fragment of 500 Kb absent in the pig. Porcine BAC spanning each breakpoint are represented with anchoring of their ends on the human genome. Porcine BAC defining at best the localization of each breakpoint are shown with anchoring of their ends on the human genome.

To confirm the reality of the rearrangements identified during the building of the BAC contig, we tried to identify a porcine BAC spanning each of the 4 breakpoints, with BES anchored on the 2 expected locations on human genome (Figure 3). In order to increase the probability of identifying these BAC, we also selected from the international map [17] BAC overlapping INRA BAC close to synteny breaking points. For BAC with no significant homology, we used FASTA to detect any potential homology, using less stringent parameters. In pig, one BAC anchored on the 2 expected human locations was identified, for the 4 breaking points A-B, C-D, E-F and G-H (bI0149A04 (33.03 Mb – 55.93 Mb), bT0141E11 (53.48 Mb-62.48 Mb), bI0222C06 (63.7 Mb – 57.85 Mb) and RPCI44_0428O15 (56.20 Mb – 33.35 Mb)) (Figure 3).

Analysis of the anchorage on the human map of all BES available in these regions, allowed us to estimate more precisely the location of the 4 breakpoints on the human genome (Figure 3): A/H breakpoint is located at 33.26 – 33.35 Mb, I/C at 53.24 – 53.48 Mb, B/G at 55.93 – 56.01, F/D after 58.03 Mb and before 62.46 Mb (with human centromere located in this interval), E point after 63.78 Mb.

### Multispecies comparison of the organization of the studied region

To compare the order of genes in this region between various species, we identified the location on the genome of sequenced species of several genes representing each chromosomal fragment described previously: fragment 1 (*RXRb, RING1*), fragment 2 (*VPS52, RPS18 *and *RAB2L*), fragment 3 (*ELOVL5*), fragment 4 (*GCLC, LANO, TINAG *and *BMP5*), fragment 5 (*COL21A1, DST *and *PRIM2A*), fragment 6 (*KHDRBS2*) and fragment 7 (*GLULD1*). Their locations were determined by blasting the porcine sequences against current genome assemblies: mouse sequence Build 33 assembly from NCBI, dog whole genome shotgun (WGS) assembly v1.0 (July 2004), and chicken galGal2 assembly. To enrich the set of data available for the chicken genome, we developed markers for *VPS52 *and *RAB2L *which were not identified on the sequence of chicken genome with the ESTs available for these genes. We mapped these 2 markers on chickRH panel [20] and analyzed the results on ChickRH server [21].

It was also important to include a species phylogenetically close to the pig in the comparison. We selected cattle, a species for which both detailed comparative mapping studies have been performed [22,23] and a partial genomic sequence is available. However, in the region of interest, the assembly of the bovine genome is by no means complete (each gene is found in a different scaffold after blasting the sequence of genes mapped in pig against the bovine genome) and available RH maps are insufficiently detailed. This led us to develop a bovine RH map in this region. We used the ICCARE browser [13], to select bovine EST available for 16 genes of this region: *RXRb, RING1, HKE4, HSD17B8, VPS52, RAB2L, ELOVL5, TINAG, LANO, COL21A1, DST, BAG2, PRIM2A, KHDRBS2, PHF3 *and *PTP4A1 *(Table 2). We developed pairs of primers for each gene and we mapped them on the RH5000 bovine panel [22]. An average retention frequency of 36% was observed, ranging from 26% for GLO1 to 50% for *PTP4A1*. Vectors obtained for the 16 new genes were analyzed together with 31 markers previously available for BTA23 [22]. A 1000:1 framework map including 10 genes, was built with Carthagene software [14] in the region from *PRIM2A *to *VPS52 *(Figure 1B). In addition gene *KHDRBS2 *is linked with a LOD score 3.5 at the top of this map. Four additional genes *HSD17B8, RXRb, RING1*, and *HKE4 *were found totally linked and mapped at their most likely location relatively to markers of the framework (Figure 1B). *ELOVL5 *was found linked to markers previously mapped in a distal position. *PHF3 *and *PTP4A1 *were found significantly linked together (LOD = 13.2), but totally unlinked to other genes.

**Figure 4:**
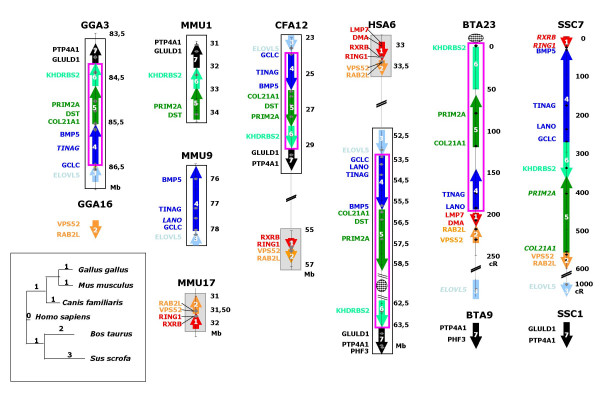
**Multi-species (*Gallus gallus, Sus scrofa, Bos taurus, Canis familiaris, Mus musculus *and *Homo sapiens*) comparative map**. Data concerning *Canis familiaris, Gallus Gallus, Mus musculus*, and *Homo sapiens *result respectively from Dog Jul. 2004 (canFam1) assembly, Chicken Feb. 2004 (galGal2) assembly, mouse Build 33 assembly by NCBI and human NCBI build 35. Data on chicken GGA 16 were not available on genome assembly, and were produced on ChickRH panel [20]. Maps concerning *Sus scrofa *and *Bos taurus*, are 1000:1 framework maps produced on RH panel (see Figure 2). Markers written in bold belong to the framework map and markers in italic are mapped at their most likely location. The different arrows represent conserved synteny group. White boxes framed in black represent fragments conserved between *Gallus gallus, Canis familiaris, Mus musculus *and *Homo sapiens*. Boxes framed in pink represent fragments conserved between *Gallus gallus, Canis familiaris, Mus musculus*, *Homo sapiens *and *Bos taurus*. Grey boxes represent fragments conserved between *Canis familiaris, Mus musculus *and *Homo sapiens*.

In the dog genome, all the selected genes are grouped into two large fragments (fragments 3, 4, 5, 6 and 7 and fragments 1 and 2) located on chromosome 12 (Figure 4). In the mouse genome, the same region is similarly split into 3 conserved fragments (Figure 4): fragments 5, 6 and 7 are located on MMU1; fragments 3 and 4 on MMU9; and fragments 1 and 2 on MMU17. In chicken, a conserved fragment containing fragments 3, 4, 5, 6 and 7 is located on GGA3. Two other genes, *VPS52 *and *RAB2L *(fragment 2), are mapped in a linkage group that also contains markers derived from chicken MHC region located on GGA16 (Figure 4). No information is available for *RXRB *and *RING1 *(fragment 1). If the order of genes is thus globally conserved in these species, a very different situation is observed on the bovine genome (Figure 4): 14 of the 16 selected genes are localized on BTA23, but in an order very different from the generally conserved order, and 2 others genes (*PHF3 *and *PTP4A1 *representing fragment 7) are located on chromosome 9.

The analysis of these results shows that the whole fragment (comprising fragments 4, 5 and 6) inserted on the pig genome between fragments 1 and 2 (*RING1 *and *VPS52*) seems very well conserved in chicken, dog, mouse and human. In cattle and pig, belonging both to the clade of *Cetartiodactyles*, fragments 4, 5 and 6 are found at a different location. However, in this 5.2 Mb large fragment, a total conservation of gene order was observed between human and cattle genomes. A global analysis of the chromosomal rearrangements that occurred during evolution was carried out using GRIMM. [24]. The results obtained are summarized on Figure 4.

**Table 2:** Bovine markers developed for RH mapping

**STS-EST**	**SEQUENCE UP**	**SEQUENCE DN**	**SIZE OF PCR FRAGMENT**	**% RETENTION ON RH5000**	**POSITION (cR)**	**HUMAN LOCATION (Mb)**
**KHDRBS2**	ATGGGGTTGCAAACAAGAAG	TTTGACAAATTGTGGTTTCCT	300	36	0	62.75
**PRIM2A**	AAAGCAGCATTCTGGACTGG	CCAGAAAAGGTCTGGGTCAA	104	40	82	57.29
**BAG2**	AATCTGACTGCGAACCGTCT	GTTTCCCAGGTCATCCAGAA	150	36	94	57.15
**DST**	CCACCTGGACCTGAGACCTA	ACAGGCGCTACTGTCCACTC	101	39	106	56.58
**COL21A1**	CAGTTCTTCGGAGCCTTGAG	GACTGCAACTGACTGGCTGA	133	34	125	56.12
**TINAG**	ACCGCATAGCAATTCAGTCC	ACCACCAAGCCCTATCAACA	123	31	184	54.28
**LANO**	CGGTCTCCAGCTTGATTGTT	TCCAAGAGAAGGTGGGTTTG	185	37	202	53.77
**RAB2L**	TCAATCTGGTCCCCTCTCAG	CCAGGACAGAGCTGACCACT	141	33	230	33.37
*B3GALT4*	GGAGGCAAGCACCAGGTAT	CTGGCCACCTTCAGGATAAG	125	37		33.35
*RING1*	ATCCAAAGTGACCCCACAAG	GGCAATAGAAAAGGCAGCAG	228	37		33.29
*HKE4*	GACTATGTCCGTGGTGCAAA	CTGTACGAAGACCTGCACGA	181	37		33.28
*RXRB*	CTGAAAAAGGTGGTGGTGGT	CATAAGAGCCGCAGAGAACC	193	37		33.27
*VPS52*	ACCCAGCTGATCCAGCTCTA	ATCAGAAGTTGGGCTTGTGC	138	29		33.33
*ELOVL5*	TCATGCTTTGATTTTGCACAT	TGTAAACACGAAGCCGTGACT	104	28		53.28
*PTP4A1*	GTGATTCCCAGCCTCTCTTG	CCACTTGTTCCCGGACAGTA	199	50		64.35
*PHF3*	CCATTTCGAAGAGGATCAGC	AACCCAGGTGGGCTAGACTT	188	37		64.45

Markers in bold belong to the framework RH map whereas markers in italic are positionned with the best likelihood. Primer sequences, retention fraction, RH map location on bovine map and human location are indicated.

## Discussion

The study of porcine chromosome 7 is of particular interest due to the presence of many QTL affecting traits of economic importance and of MHC [1,2]. One approach to study this chromosome is to identify all the genes located on this chromosome and to compare their order relatively to that in the human genome. Previously, a strong conservation of gene order was found between a large fragment of porcine SSC7 and human HSA6 p arm [9,10]. We had highlighted the existence of a fragment estimated to be less than 4 Mb rearranged between SSC7 and HSA6 [11]. This fragment defined by 3 genes (*LANO *previously named *FLJ10775*, *BMP5 *and *BAG2*) located on HSA6p12.1-6p11.2 is found in the pig genome between genes located on HSA6p21.32 [11]. The building of a BAC contig in the chromosomal region surrounding SLA complex provided additional information, even if the contig remained incomplete [12]. This study describes a situation that is even more complex, suggesting the presence of *VPS52 *(previously named *SACM2L*) within the rearranged region. In the pig genome, this rearranged chromosomal region is included in the region where the QTL of interest is mapped, between markers Sw1856 and *NFY *(Demeure et al, JAS, in press). In order to identify all candidate genes that may be responsible for the QTL effects, it was particularly important to establish a detailed comparative mapping analysis of this region.

We first built a dense porcine RH map (Figure 1A) using the high resolution IMNpRH2_12000rad _panel, allowing us to order unambiguously genes at distances of 100 to 600 Kb [7]. Our data confirmed the shift of the rearranged fragment and its precise location in the pig genome between *RING1 *and *VPS52 *genes (previously called *SACM2L*) (fragments 1 and 2). In contrast to Barbosa et al [12], we show that *VPS52 *is located close to *RAB2L *in both species. We also determined more precisely the extent of the fragment rearranged between the human and pig genomes. The upper frontier of this fragment is located between *ELOVL5 *(previously named *HELO1*) and *GCLC*, located respectively at 53.28 and 53.43 Mb on HSA6. The lower boundary of the fragment is defined by the first gene located on HSA6q also mapped on SSC1. This first gene was previously designated as *FLJ13159*, and was located at 71.53 Mb on HSA6 [11]. We showed that *GLULD1*, located at 64.05 Mb on HSA6, also mapped on SSC1 and constitutes the limit currently identified in this fragment.

The gene-based RH map (Figure 1A) that we built allowed us to define 3 sub-fragments of this region arranged differently on the pig and human genomes. Two of these sub-fragments are delimited by genes: *BMP5 *and *GCLC *for the first one (fragment 4), and *PRIM2A *and *COL21A1 *for the second (fragment 5). No genes were identified in the third fragment (fragment 6), which contains the anonymous microsatellite Sw1856. This map greatly refines the internal rearrangements suggested by our previous work [11]. The sum of the sizes of fragments 4 and 5 is ~4.3 Mb and is similar to the orthologous human region. Fragment 6 covers 1.4 Mb in the human map, whereas the size of this fragment in the porcine RH map suggests a size of approximately 900 Kb which would indicate that a fragment of 500 Kb is absent in the porcine genome. The fragment containing Sw1856 on the pig genome might thus be orthologous to part of the region flanking the centromere of HSA6, but at that step it remained to be proved (Figure 1A).

To confirm the order of genes suggested by RH mapping and study this region in more detail, we built a contig of BAC covering the entire region (Figure 2). The availability of elementary contigs resulting from the fingerprinting of clones from the INRA BAC library and sequences of extremities of 35000 of these BAC (BES) (Chardon et al, in preparation) greatly facilitated the establishment of this complete contig. Our work completes the studies begun by Barbosa et al [12], who presented 4 small contigs. The analysis of porcine BES anchoring on the human genome demonstrates that the fragment 6 containing porcine marker Sw1856 corresponds to a fragment framing the centromere of HSA6 (6p11.1-6q11.1). Taking into account the average distance between two hits of porcine BES on human genome, we can say that if additional fragments from other human chromosomes are located in this rearranged region of the porcine genome, they should measure <50 Kb.

Analysis of the anchoring of porcine BES on the human genome highlighted also additional results: 1) It was indeed possible to define the position of the 3 synteny breakpoints (Figure 3). On the human genome, these synteny breakpoints (A-H, I-C and B-G) are situated between genes *RING1*-*VPS52*, *ELOVL5*-*GCLC *and *BMP5*-*COL21A1*. Each of these 3 synteny breakpoints are localized in a interval of ~100–200 Kb, between the human positions 33.26 Mb-33.35 Mb, 53.24 Mb-53.48 Mb and 55.93 Mb-56.01 Mb. 2) The fourth synteny breakpoint (E-F on the pig genome and F-D on the human genome) was identified thanks to the anchoring of the BES in the human genome: the two fragments framing HSA6 centromere were found in a different order on the pig genome without the presence of a centromere (Figure 3). 3) For several BES, alignment on the human genome showed 2 possible positions with similar likelihood. This result suggested the existence of duplicated sequences close to the human centromere, which was confirmed by a dot plot on itself of the sequence of this human region. Phylogeny studies of human chromosome 6 demonstrated the repositioning of this centromere among primate species in a region equivalent to 6p22.1 in *Eulemur macaco*, *Callithrix jacchus *and *Lagothrix lagothrica*, to 6q22 in *Macaca fascicularis *and *Presbytis cristata*, and to 6p12 in the great apes [25]. The repeated sequences we highlighted might then correspond to sequences of an ancestral centromere positioned in HSA6p22.1 [25]. 4) Lastly, *in silico *analysis of the BES alignments on the human genome provided evidence of the absence from the pig genome of a fragment of ~400–500 Kb found in human genome. One can argue that the 2 BES of BAC bI0688H06 match on the human genome with sequences distant of 650 Kb (at 63.02 Mb and 63.67 Mb). The absence of anchoring of other BES between 63.05 Mb and 63.55 Mb, whereas one hit is found for each 20 Kb in the surrounding region, confirmed that this fragment is absent from the pig genome.

This region appears to be heavily rearranged between the porcine and human genomes. We wanted to determine if these rearrangements are specific of suines or *Cetartiodactyles*, or occur in many other species. To identify gene order in other species, we used the genomic sequence when available. For *Gallus gallus*, the assembly of the genome has not been totally completed; this is why we enriched information obtained from the sequence by RH mapping data when it was necessary (Figure 4). The order of genes seems to be conserved between the chicken and human genomes except for the *RXRb *and *RING1 *genes (belonging to fragment 1). These 2 genes which may not exist in the chicken genome are located in other species in the extended class II region of MHC. The chicken MHC, named the B locus, is a 92 Kb DNA sequence containing 19 genes [26,27]. It defines a minimal essential set of MHC genes conserved over 200 million years of divergence between birds and mammals [27].

As the available assembly of the cow genome consists of many independent sequence scaffolds, we determined the gene order by RH mapping [22] (Figure 1B). Our results reveal that genes from *GCLC *to *KHDRBS2 *(fragments 4, 5 and 6) are similarly ordered on the human and bovine genomes in this region, which suggests that the internal rearrangements of these 3 fragments, comparing the human and porcine genomes, are specific to the evolution of the suine family. Everts-van der Wind et al. [18] reported that 12 cattle chromosomes are entirely similar to a complete human chromosome arm, and in particular, that p and q arms of HSA6 are homologs of BTA23 and BTA9. Our study modifies slightly this report, in that *KHDRBS2 *(belonging to fragment 6), the first gene localized on the long arm of human chromosome 6, is probably located on bovine chromosome 23. As the number of genes in this region, as in other species, is very limited; it is difficult to develop and map markers in this region. The end of BTA23 might thus signal the location of the centromere in the ancestors of *Cetartiodactyles*, in which the fission between the 2 chromosomal arms occurred. In this region, a further comparison of the bovine genomic sequence with the human sequence will be of interest.

We compared the gene order in the pig (*Sus scrofa*) and cattle (*Bos Taurus*) with the situation in the genomic sequence of *Mus musculus*, *Canis familiaris*, *Gallus gallus *and *Homo sapiens *(Figure 4). We observed at first that on the pig genome, a large fragment (comprising fragments 4, 5 and 6) of ~5.2 Mb is shifted between *RING1 *and *VPS52*, 2 genes belonging to the extended class II region of the MHC. If MHC is one of the most dynamic regions of the genome [28], a high level of conservation of genome organization provides evidence in this region of extended class II genes [29]. Fourteen genes occur in this region between *RING1 *and the *VPS52 *in dog, cat, human and mouse genomes, which only differs in the presence of processed pseudogenes [29]. This small fragment of 500 Kb containing *RING1 *and *VPS52 *is thus dynamically involved in chromosomal evolution. It is interesting to note that the translocation of the 5.2 Mb fragment occurs in this region. In this region, the genomic organization of the fragment and its flanking regions is similar in mouse, human, dog and chicken, whereas translocation is observed in pig and cattle. This suggests that this genomic reorganization occurred after the divergence of the common ancestor of *Cetartiodactyles *from the *Ferungulates *ancestor between 94 to 62 million years ago [30]. The gene order observed in pig for the 5.2 Mb fragment is different from the one identified in other species, indicating that these internal rearrangements occurred after ruminant-suine speciation. We identified a situation really different from the map presented by Everts-van der Wind et al. for the proximal part of BTA23 close to the centromere [23]. Further study of the rearrangements that occurred in ruminants in this region of extended class II of the MHC complex is clearly needed.

We used GRIMM to compare the different maps available and to present a possible evolution of this region between the various species [24]. The unrooted tree we obtained (Figure 4) presents a very different view from a classical phylogenic tree [31,32], highlighting a high rate of rearrangements in this region that have occurred recently in *Cetartiodactyles*. The study of local rearrangements also provides a view different from the recent multispecies comparison of this region [33]. In order to filter out noise induced by mapping errors, this analysis from Murphy et al only took into account fragments >3 Mb, defined by three genes. Among other results, they documented the reuse of evolutionary breakpoint regions identified between conserved homologous synteny blocks. Our study indicates that such a study should be redone on a more detailed scale, when the accuracy of mapping data is adequate.

## Conclusion

A QTL affecting fattening and growth had been mapped in the porcine region SSC 7q1.2. As we had shown that a small fragment is translocated between porcine and human genome, it was important to establish a final comparative map of this region. The RH map and the BAC contig we developed in this region allowed us to attest that the translocated fragment is approximately 5.2 Mb long. It consists in three blocks, found in HSA6 between 53.45 and 63.5 Mb, one of these blocks containing a few genes, framing the centromere on the human chromosome. The multispecies study allowed us to confirm that the translocation of this 5.2 Mb fragment occurred in the common ancestor of pig and ruminants, whereas internal rearrangements are found only in the pig genome. The exact knowledge of the genomic content of this region permits us now to establish a list of all possible candidate genes that may affect the growth and fattening of pigs.

## Methods

### Markers and PCR amplification

The ICCARE (Interspecific Comparative Clustering and Annotation foR Ests) tool available at [13] allowed us to select 16 genes in the region between *RXRB *and *RAB2L*, for which a porcine EST sequence is available (Table 1). In addition, 88 markers have been developed from INRA BAC ends. Several result from personal sequencing using the BigDye Terminator V3.1 cycle sequencing kit (Applied Biosystems) on the ABI 3700 automatic sequencer; these sequences were submitted to EMBL Nucleotide Sequence Database (AM156864 to AM156903) [34]. Others come from BAC ends sequences available on ENSEMBL [19]. Primers have been chosen using Primer3 software [35]. PCR was performed in a 15 μl reaction volume containing 25 ng template DNA, 200 μM dNTP, 0.25 μM specific primer pair and 0.5 U Taq polymerase (GoTAQ) in the reaction buffer supplied by the manufacturer. Amplifications were carried out on a Gene-Amp System 9700 (Applied Biosystems) thermocycler. Thermal cycling parameters were defined as follows: denaturation at 94°C for 5 min, followed by 32 cycles of (1) 94°C for 45 sec, (2) annealing temperature for 45 sec, (3) 72°C for 45 sec, and a final extension was performed at 72°C for 5 min. PCR products were analyzed on a 2% agarose gel, and visualized after ethidium bromide staining.

### RH mapping

Markers were mapped on the IMpRH panel [6] and IMNpRH2 panel [7] according to the INRA protocols. For markers used on IMpRH panel, vectors were submitted to the IMpRH web server at [36] for an initial two-point assignment. Then, vectors obtained on IMpRH and IMNpRH2 were analyzed with Carthagene software [14]. A 1000:1 framework map was built with buildfw option using a stepwise locus adding strategy under an haploid model of fragment retention. The different provisional frameworks were checked using the simulated annealing algorithm to test inversion of the map fragments, and the flips algorithm to test all local permutations in a window of 6 markers. The resulting RH map was drawn with Map-chart 2.0 [37]. An RH map was also established for the bovine genome using the bovine RH panel RH5000 [22]. Several markers were also mapped on the chicken genome using ChickRH6 panel [20].

### BAC screening and chromosome walking

The INRA BAC porcine library [12], was screened by PCR on BAC super pools and pools. The presence of the expected sequence in the identified BAC was checked by PCR on DNA from the isolated BAC clone. For the selected BAC that had been previously fingerprinted and incorporated in a primary INRA contig (Chardon et al in preparation), all BAC from this contig were selected from INRA BAC Server [38]. To confirm the BAC overlapping, markers developed from BES were tested against DNA of BAC belonging to its primary contig or BAC located at the extremities of adjacent contigs.

### Multispecies comparison

In order to determine genes order in other species, 12 genes (*RXRB*, *RING1*, *VPS52*, *RAB2L*, *ELOVL5*, *GCLC*, *BMP5*, *COL21A1*, *DST*, *PRIM2A*, KHDBRS2, *GLULD1*) were located with Blast in human by using NCBI build 35 produced by the International Human Genome Sequencing Consortium, in mouse (*Mus musculus*) draft genome data was obtained from the Build 33 assembly by NCBI, in chicken (*Gallus gallus*) draft assembly was produced by the Genome Sequencing Center at the Washington University School of Medicine in St. Louis, in dog (*Canis familiaris*) whole genome shotgun (WGS) assembly v1.0 was sequenced and assembled by the Broad Institute of MIT/Harvard and Agencourt Bioscience. We used the GRIMM software [24] to estimate the rearrangements between species and build a multispecies phylogenetic tree.

## List of abbreviations

MHC major histocompatibility complex; QTL quantitative trait locus; SLA swine leucocyte antigen; RH radiation hybrid; BAC bacterial artificial chromosome; BES BAC end sequence, cR centi Ray; HSA human chromosome; SSC porcine chromosome; MMU murine chromosome; BTA bovine chromosome; GGA chicken chromosome; CFA dog chromosome; IMNpRH2 INRA Minnesota Nevada porcine Radiation Hybrid panel 2.

BAC bIxxxZxx are from INRA BAC library [12], BAC bExxxZxx are from CHORI242 library (P. de Jong, et al., unpubliched), BAC bTxxxZxx are from Roslin BAC library [39], RPCI44_xxxZxx are BAc clones from RPCI44 library (P. de Jong et al., unpublished).

## Authors' contributions

JD built the complete contig using the INRA BAC library fingerprinting, did the genotyping of the porcine and bovine RH panels, made the *in silico *analyses and drafted the manuscript. JR supervised this work. KF took part in the development and the explanation of the techniques. MG provided DNA of bovine RH5000 panel and the vectors for the bovine markers previously mapped in the studied region. MM did the genotyping and the analyses on the chicken RH panel. CR performed some preliminary BAC screening. PC coordinated the fingerprinting of INRA BAC and the providing of all BAC necessary to the construction of the contig. DM conceptualized the investigation, analyzed the RH data, and finalized the manuscript.
